# Method for evaluating prediction models that apply the results of randomized trials to individual patients

**DOI:** 10.1186/1745-6215-8-14

**Published:** 2007-06-05

**Authors:** Andrew J Vickers, Michael W Kattan, Daniel Sargent

**Affiliations:** 1 Department of Epidemiology and , Memorial Sloan-Kettering Cancer Center, 1275 York Avenue NY, NY 10021, USA; 2 Department of Quantitative Health Sciences, Cleveland Clinic, 9500 Euclid/Wb4, Cleveland, OH 44195, USA; 3 Division of Biostatistics, Mayo Clinic, 200 First Street SW, Rochester, MN 55905, USA

## Abstract

**Introduction:**

The clinical significance of a treatment effect demonstrated in a randomized trial is typically assessed by reference to differences in event rates at the group level. An alternative is to make individualized predictions for each patient based on a prediction model. This approach is growing in popularity, particularly for cancer. Despite its intuitive advantages, it remains plausible that some prediction models may do more harm than good. Here we present a novel method for determining whether predictions from a model should be used to apply the results of a randomized trial to individual patients, as opposed to using group level results.

**Methods:**

We propose applying the prediction model to a data set from a randomized trial and examining the results of patients for whom the treatment arm recommended by a prediction model is congruent with allocation. These results are compared with the strategy of treating all patients through use of a net benefit function that incorporates both the number of patients treated and the outcome. We examined models developed using data sets regarding adjuvant chemotherapy for colorectal cancer and Dutasteride for benign prostatic hypertrophy.

**Results:**

For adjuvant chemotherapy, we found that patients who would opt for chemotherapy even for small risk reductions, and, conversely, those who would require a very large risk reduction, would on average be harmed by using a prediction model; those with intermediate preferences would on average benefit by allowing such information to help their decision making. Use of prediction could, at worst, lead to the equivalent of an additional death or recurrence per 143 patients; at best it could lead to the equivalent of a reduction in the number of treatments of 25% without an increase in event rates. In the Dutasteride case, where the average benefit of treatment is more modest, there is a small benefit of prediction modelling, equivalent to a reduction of one event for every 100 patients given an individualized prediction.

**Conclusion:**

The size of the benefit associated with appropriate clinical implementation of a good prediction model is sufficient to warrant development of further models. However, care is advised in the implementation of prediction modelling, especially for patients who would opt for treatment even if it was of relatively little benefit.

## Introduction

The typical approach to interpreting and applying the results of a randomized trial to clinical practice is to determine first whether differences between groups are statistically significant, and then second, whether they are clinically significant, that is, large enough to be worthwhile. Clinical significance can be evaluated in two distinct ways. In some cases, where treatment is associated with side-effects or is inconvenient, clinical significance is a matter for the individual patient. A good example is adjuvant chemotherapy for early stage breast cancer. Patients vary as to how unpleasant they rate the various side-effects of chemotherapy relative to the importance of avoiding an early death, and different patients will therefore disagree whether a certain reduction in the risk of death is worthwhile. Clinical significance can also be evaluated from a clinician or policy maker perspective: a clinician might evaluate the additional benefit of a new analgesic in the light of an increased risk of a rare, fatal side-effect; a policy maker might consider whether the benefit provided by an expensive treatment, such as beta-interferon for multiple sclerosis, is worth the cost.

Clinical significance is typically assessed by reference to between-group differences in means or proportions. In the adjuvant chemotherapy example, the result of a trial or meta-analysis might be that 35% of breast cancer patients receiving adjuvant chemotherapy died within 10 years compared to 40% of the controls undergoing surgery alone. A doctor practicing evidence-based medicine might therefore counsel a breast cancer patient that she reduces her risk of death by 5% if she undergoes chemotherapy. The patient would then consider whether this is sufficient benefit to pursue treatment.

Implicit in the use of such group level estimates is that all patients are at average risk and have an average likelihood of response to therapy. This is known to be untrue: a woman with a large breast tumor that has spread to local lymph nodes is at far higher risk of death from cancer than a woman with a smaller tumor and no nodal spread; moreover, response to chemotherapy depends on estrogen-receptor status.

An alternative to the group level approach to evaluating clinical significance is to make individualized predictions for each patient based on a statistical model. In the case of adjuvant chemotherapy for breast cancer, there are good data showing how both overall survival and response to chemotherapy are affected by prognostic factors such as size and grade of tumor, age, number of affected nodes and estrogen-receptor status. Given particular values of these variables for an individual patient, a prediction model can be used to predict risk of death both with and without adjuvant therapy. The difference between these two predictions is the individualized estimate of treatment benefit.

This model-based approach to individualizing estimates of treatment benefit is apparently growing in popularity. This is particularly true for cancer, where both the benefits and harms of treatment can be large. Recent years have seen the publication of papers describing the development of prediction models for treatment benefit [1-4] and the concomitant introduction of web-based systems [5-7] that enable complex predictions to be made with a few keystrokes. There are clear indications that patients and clinicians like these models, with emerging examples of physicians explicitly recommending their use during the clinical consultation[8].

Models that predict the outcome of different cancer treatment options are intended to be used in patient counseling, that is, the clinician presents an individualized estimate of benefit to the patient and the patient then decides whether the magnitude of benefit is sufficient to pursue treatment. A slightly different use of prediction models is particularly common in cardiovascular medicine. Many therapies used to treat cardiovascular disease have competing effects: warfarin reduces the risk of embolic stroke but increases the risk of hemorrhagic stroke. Prediction models have been advocated to ensure that, for an individual patient, the decrease in the risk of the targeted cardiovascular event is greater than the risk of the cardiovascular side-effect[9,10]. Prediction models have been published for several other cardiovascular treatments[3,4]

Despite their intuitive advantages, it remains plausible that some prediction models may do more harm than good. For example, it is possible that a model might mistakenly predict little or no benefit from adjuvant chemotherapy for a patient with a small number of viable tumor cells after surgery. The patient forgoes treatment and dies from a curable cancer.

The current approach to determining whether a prediction model is of clinical value is implicit and indirect: researchers address whether their model has good properties in general using measures of predictive accuracy such as calibration and discrimination, and more qualitative criteria such as validation on an independent data set and the size and variability of the data used to generate the model. A key drawback of this approach is that predictive accuracy does not fully determine the clinical value of a model: it is quite possible for an accurate model to be useless, and a model with relatively poor accuracy to be useful[11]. For example, no one would consider a using a model to predict response to a highly effective, low-risk treatment for a mortal disease (such as antibiotics for bacterial meningitis); conversely, a model of moderate accuracy may be useful if the benefit of treatment was marginal.

In this paper, we develop a decision-analytic approach[11] for determining whether a prediction model should be used to apply the results of a randomized trial to individual patients. We propose that our methods be used by researchers who develop prediction models. Our methods would supplement traditional measures, such as the area-under-the-ROC-curve, to help potential users determine the value of the modelling approach.

## Methods

### Notation

We define D as the predicted difference in outcome between two treatment alternatives: D_i _is the individualized difference that varies between patients on the basis of a prediction model; D without a subscript is the group level estimate derived, for example, from the difference between event rates in a randomized trial. We define T in terms of the threshold for agreeing to treatment: T without subscript is a single threshold applied to all patients; T_i _is an individualized threshold that differs from patient to patient based on personal values and circumstances. T is defined so that we opt for treatment if T < D and avoid treatment if T > D. If T = D, then we are in a state of equipoise and are unsure as to whether treatment is worthwhile.

Most of our discussion will refer to the binary case, where patients are at risk for an event ("event"), such as death or a recurrence of cancer. In this case both T and D are expressed in proportions: for example, if the death rate in a trial was 10% in controls and 7% in the treated group, D would be 3%. Nonetheless, the same notation can be used for a continuous endpoint, such as a pain score, or length of survival in days.

Our notation has an immediate application for categorizing modes of decision making. In paternalistic medicine, or in guidelines based on expert opinion, D is compared with T. Evidence-based counselling of individual patients compares D with T_i_. This paper is concerned with comparison of D_i _with T_i_, patient counselling based on individualized risk prediction accounting for patient-specific preferences as described in the chemotherapy example, and comparisons of D_i _with T, where individualized predictions of treatment benefit are compared to a fixed threshold, such as in the cardiovascular medicine example.

### Prediction Modelling

Several different methods of developing prediction models to inform treatment decisions have been described. These include using the randomized trial data to develop the model[1] or creating a model for untreated patients on the basis of cohort studies, then using a fixed relative risk derived from a randomized trial to estimate risk in treated patients[2,10]. Models also differ in terms of the specific statistical techniques applied, for example, use of non-linear terms, bootstrap correction or model selection criteria. For the purposes of this paper, we will remain agnostic about the methods of model building, except that investigators need to be able to demonstrate the validity of their model independent of the methods we propose. This would include documentation of the data and methods used to build the model, measures of model accuracy, such as the concordance index[12], and correction for overfit.

### Method for evaluation of prediction models: background theory

We propose an extension of decision-curve analysis, a previously published method for evaluating prediction models[13]. In its original formulation, decision-curve analysis was used for models that predicted the probability of an event, such as the presence of cancer outside the prostate capsule in a patient scheduled for prostatectomy. Here we use the method to predict the difference between the probabilities of an event under two conditions: treatment and control.

We start by noting that the value of a prediction model is usually evaluated by applying it to a data set and determining whether the predictions match the outcomes actually observed. For our current purposes, we are not interested in the predictions themselves, but in the decisions that result from these predictions. We therefore require a data set in which there is a decision between two treatments and a subsequent outcome. Randomized trials provide such a data set, except that the "decision" of which treatment to use is made by chance. Nonetheless, a randomly chosen treatment will be congruent with how a decision would have been made on the basis of the prediction model for a proportion of patients in the trial. Our proposal is therefore that the prediction model be applied to a data set from a randomized trial or meta-analysis and the results documented for patients whose randomized allocation is congruent with the recommendations of the prediction model. These results could then be compared with use of a group level estimate.

To make this comparison, we note that there are three strategies for applying the results of a randomized trial to clinical practice: treat all patients (the typical approach where treatment effects are sufficiently large), treat no patients (where the difference between groups is not thought to be clinically significant), and treat patients according to a prediction model. Applying each of these strategies to a group of patients will lead to certain number being treated and a certain number experiencing the study event. For example, take a hypothetical trial of adjuvant chemotherapy with 2000 patients where the death rates were 35% and 40% for patients on treatment and control, respectively. Applying the strategy of "treat all" to 1000 patients, would result in 1000 treatments and 350 events; treating no-one would result in 400 events. Applying a prediction model, where only patients with a predicted benefit of T or more are treated, typically leads to a number of treatments and a number of events that is intermediate. For this hypothetical example, let us assume that the prediction model leads to 650 treatments and 355 events; the prediction model leads to some patients not being treated as not all have estimated D_i_'s less than T, but some patients who would benefit from chemotherapy do not undergo chemotherapy and therefore relapse. Hence, compared with treating all patients, the use of a prediction strategy will tend to increase the number of events.

We propose comparing the "net benefit" of the two strategies involving treatment: treat all patients vs. treat according to the prediction model. Net benefit is a concept often used in economic analysis and is simply benefits minus harms. In the case of a treatment, "benefits" are associated with reduction in the event rate compared to no additional treatment: in an adjuvant therapy trial, for instance, benefit would be a reduction in cancer recurrences or deaths compared to surgery alone. "Harms" are associated with the treatment itself: side-effects, risks, costs, inconvenience and so on.

To calculate net benefit we require a single scale for treatments and events. We have previously demonstrated[13] that this question -"How many treatments are equivalent to one event?" – is answered by the threshold at which a patient would opt for treatment, that is, T. We know from clinical practice that patients will demand that a treatment with important side-effects must lead to a relatively larger reduction in the risk of event than a treatment with trivial toxicities. In the appendix (see additional material file 1), we demonstrate that T is equivalent to the ratio between harms of treatment and those of an event. Thus, if a patient states that they would be unsure what to do if the benefit of treatment were an absolute 5% risk reduction, they are telling us that they consider an event to be about 20 times worse than the risks, side-effects and inconvenience of treatment.

### Method for evaluation of prediction models: calculation of net benefit

We calculate net benefit as the decrease in the proportion of events associated with treatment minus the proportion of patients treated multiplied by T. That is, we combine treatments given and events by weighting the proportion of treatments by the ratio of harm from treatment and harm from event. The unit is therefore in terms of events, or, alternatively, the disutility of event is defined as 1.

Net benefit = decrease in event rate – treatment rate × T

More generally, we define net benefit as:

∑i=1i=n0x0in0−∑i=1i=n1x1in1−n1Tn1+n0
 MathType@MTEF@5@5@+=feaafiart1ev1aaatCvAUfKttLearuWrP9MDH5MBPbIqV92AaeXatLxBI9gBaebbnrfifHhDYfgasaacH8akY=wiFfYdH8Gipec8Eeeu0xXdbba9frFj0=OqFfea0dXdd9vqai=hGuQ8kuc9pgc9s8qqaq=dirpe0xb9q8qiLsFr0=vr0=vr0dc8meaabaqaciaacaGaaeqabaqabeGadaaakeaadaWcaaqaamaaqahabaGaemiEaG3aaSbaaSqaaiabicdaWiabdMgaPbqabaaabaGaemyAaKMaeyypa0JaeGymaedabaGaemyAaKMaeyypa0JaemOBa42aaSbaaWqaaiabicdaWaqabaaaniabggHiLdaakeaacqWGUbGBdaWgaaWcbaGaeGimaadabeaaaaGccqGHsisldaWcaaqaamaaqahabaGaemiEaG3aaSbaaSqaaiabigdaXiabdMgaPbqabaaabaGaemyAaKMaeyypa0JaeGymaedabaGaemyAaKMaeyypa0JaemOBa42aaSbaaWqaaiabigdaXaqabaaaniabggHiLdaakeaacqWGUbGBdaWgaaWcbaGaeGymaedabeaaaaGccqGHsisldaWcaaqaaiabd6gaUnaaBaaaleaacqaIXaqmaeqaaOGaemivaqfabaGaemOBa42aaSbaaSqaaiabigdaXaqabaGccqGHRaWkcqWGUbGBdaWgaaWcbaGaeGimaadabeaaaaaaaa@5A38@

where *n *is the number of patients, 1 and 0 are indicators for treatment and no treatment respectively, *x *is the indicator for event, and i is an indicator for each patient. To illustrate calculation of net benefit, and as a simple proof, we will use the hypothetical data above and set T at 5% (see table 1). As T is equal to the observed difference between groups, net benefit should be and is zero for the strategy of "treat all". The "treat by prediction" model has a net benefit of 0.0125, suggesting that, for a T of 5%, prediction is the best strategy.

**Table 1:** Calculation of net benefit in a hypothetical data set for a T of 5%.

Strategy	Treat no patients, regardless of model	Treat all patients, regardless of model	Treat according to prediction model		
			Prediction model recommends treatment	Prediction model recommends no treatment	Prediction model total

Number of patients	1000	1000	650	350	1000
Number of events	400 (40%)	350 (35%)	253 (39%)	102 (29%)	355 (35.5%)
Decrease in event rate		5%			4.5%
Number of treatments		1000 (100%)			650 (65%)
Net benefit		5% – 100% × 0.05 = 0			4.5% – 65% × 0.05 = 0.0125

The results in the first and second columns give the results from the treatment and control groups. The third column shows that there were 650 patients in the treatment group for whom the prediction model estimated a benefit of 5% or more and that 253 of these died. The fourth column shows that there were 350 patients in the control group of the randomized trial with predicted benefit of less than 5%, of whom 102 died. The net benefit is the decrease in event rate minus the treatment rate × T.

The net benefit function can also be applied to continuous endpoints. In this case *x *is the endpoint, such as depression score or number of days with pain, for each patient. When a high value of the endpoint is a desirable, such as duration of survival, *x *should be replaced in the function by -*x*. For continuous endpoints, T is defined as the minimum improvement, such as a percent reduction in pain, that a patient would require before opting for treatment.

Net benefit is calculated separately for the strategy of treating all patients – in which case the event and treatment rates are simply the observed group level proportions – and for the strategy of treating patients according to the prediction model, where event and treatment rates are calculated by using the outcomes from patients whose randomized allocation is congruent with the recommendation of the prediction model. Our methodology for determining the effectiveness of a model is therefore as follows:

1. Obtain data from one or more randomized trials. The data should consist of the variables required by the prediction model, treatment assignment and an indicator as to whether the patient experienced an event.

2. Determine the number of patients and the number of events in the control and treatment groups.

3. Apply the prediction model to the data set and estimate the individualized prediction of treatment benefit, D_i_, for each patient.

4. Choose a value for the treatment threshold, T, based on consideration of the harms associated with treatment and those associated with an event.

5. Compare the estimate for D_i _with T for each patient: if D_i _> T, define patient as "Treatment recommended"; if D_i _< T define patient as "Treatment not recommended".

6. Identify all patients where the treatment recommendation is the same as the randomized assignment. For example, if T is 3%, and a patient in the treatment arm has an estimated D_i _of 8%, the patient's actual and recommended treatment are congruent. The patient would therefore be retained for analysis. A patient with in the treatment arm with an estimate D_i _of 2%, or a patient in the control arm with a D_i _estimate of 12%, would not be included in the analysis (see table 2 for illustrative data).

**Table 2:** Hypothetical data for some example patients using a treatment threshold (T) of 5%.

ID	Group	Event	Predicted risk if treated	Predicted risk if not treated	Risk reduction from treatment (D_i_)	Treat?
1	Treatment	1	0.309	0.328	0.020	0
**2**	**Control**	**0**	**0.352**	**0.396**	**0.045**	**0**
**3**	**Treatment**	**0**	**0.474**	**0.586**	**0.112**	**1**
4	Control	1	0.219	0.350	0.132	1
**5**	**Treatment**	**1**	**0.559**	**0.704**	**0.145**	**1**
**6**	**Control**	**1**	**0.360**	**0.409**	**0.049**	**0**
**7**	**Treatment**	**0**	**0.690**	**0.884**	**0.194**	**1**
8	Control	1	0.406	0.477	0.071	1

The patients in bold are those whose treatment allocation is congruent with the recommendation of the prediction model at a T of 5%. Data from these patients would be used to calculate the outcome of decisions made by prediction modelling.

7. Determine the total number of patients with congruent treatment recommendations, the number of these who have an event and the number who are treated.

8. Apply the net benefit function to the data in 3 and 8 to determine the relative value of treating everyone and treating according to the prediction model.

9. If appropriate, repeat for a range of T's.

### Simulation studies

We first applied our methods to a variety of simulated data sets (further details are available on request from the authors). In brief our findings were that the value of prediction modelling was increased with lower event rates, less effective treatment, and higher predictive accuracy. Prediction modelling was of less benefit if event rates were high, treatment was highly effective, or predictive accuracy was poor. We also found that, even where application of a prediction model was of benefit, there were patients for whom prediction modelling should not be applied. Typically these were patients with either very high or very low T_i_: prediction modelling was often not suitable for these patients due to poor model calibration for patients at either very high or low risk, and high misclassification costs for patients with very low T_i_.

### Application to real data

We then applied our method to three real data sets. The first data set is from the ACCENT (Adjuvant Colon Cancer Endpoints) group, an international collaboration that collates and analyzes individual patient data from randomized trials of adjuvant chemotherapy for colorectal cancer. In 2004, ACCENT published a prediction model in the *Journal of Clinical Oncology*[1]*. *This model estimates the probability that a patient will be disease-free and alive at five years with and without adjuvant therapy, depending on variables such as age, stage and nodal status. The prediction tool is available online[7]. The ACCENT data consist of 3302 patients enrolled in seven randomized trials and must therefore be seen as a "gold standard" for prediction models used to implement the results of randomized trials.

Unfortunately, relatively few areas in medical research benefit from large pooled-analyses of individual patient data. To understand some of the characteristics of modelling single randomized trials, in comparison to a gold standard, we chose one of the studies ("the Moertel trial") from the ACCENT pooled-analysis[14]. A full data set for this trial is available on the Internet[15]. We modelled this data set independently of the ACCENT model and data so as to simulate the real life situation of an analyst faced with new data. The Moertel trial[14] involved a comparison between levamisole, chemotherapy plus levamisole or surgery alone in 929 patients with stage III colorectal cancer. We focused on the comparison between chemotherapy plus levamisole versus surgery alone. Details of the modelling approach used are available on request from the authors.

In the case of adjuvant therapy for colon cancer, the general recommendation is in favor of treatment, that is, the difference between the proportion of deaths in treatment and control groups is generally considered clinically relevant. In such a case, the role of prediction modelling is to identify what is likely to be a minority of patients who are at decreased risk of disease recurrence and whose expected benefit is therefore lower than the group level estimate. Alternatively, consider the case where a treatment is effective, but the difference between groups is more modest. Here the role of prediction modelling is to identify a subgroup of patients at greater than average risk whose expected benefit from treatment would more than likely outweigh the costs and harms. An example of this situation is the use of 5-alpha-reductase inhibitors to prevent complications of benign prostatic hyperplasia (BPH). Though undoubtedly effective[16], the degree of benefit appears moderate, primarily because only a small proportion of men with untreated BPH experience clinically important events such as acute urinary retention or the need for surgery[17]. The drugs can be somewhat expensive, and there is a risk of sexual side-effects. To examine the role of prediction modelling when the benefit from treatment is modest, we obtained individual patient data from three randomized trials of Dutasteride (total n = 4294) for the prevention of complications from BPH[18]. Modelling of the Dutasteride data has been previously described[19]

## Results

### ACCENT data

As previously reported, the bootstrap corrected concordance index for the prediction model for the full ACCENT data set was 0.655. There was good separation of absolute risk reduction, with the benefit of treatment ranging from a 2 – 16% improvement in 5-year disease-free survival in individual subgroups of patients. Net benefit is shown in table 3. Individualized prediction would be of clear value when the minimum benefit required for an individual patient to pursue treatment is 4 – 10%. To put this in context, in a survey of breast cancer patients[20], 50% of patients reported that it would require less than 5% reduction in absolute risk to opt for adjuvant therapy, 25% required 5 – 10%, and 25% required more than a 10% risk improvement.

**Table 3:** Net benefit for ACCENT data.

**T**	**Treat all patients**	**Treat by prediction**	**Advantage of prediction**
0.5%	0.081	0.081	0.000
1.0%	0.076	0.076	0.000
2.0%	0.066	0.060	-0.007
3.0%	0.056	0.049	-0.007
4.0%	0.046	0.050	0.004
5.0%	0.036	0.044	0.008
7.5%	0.012	0.029	0.017
10.0%	-0.014	0.013	0.013
12.5%	-0.038	-0.003	-0.003
15.0%	-0.064	-0.006	-0.006
17.5%	-0.089	0.000	0.000
20.0%	-0.114	0.000	0.000
25.0%	-0.164	0.000	0.000

T is the treatment threshold corresponding to the minimum reduction in absolute risk required to consider opting for treatment. The units are the equivalent of the number of events (death or recurrence) per patient. The "advantage of prediction" column shows the increment in net benefit of prediction compared to the better of "treat all" and "treat none".

To illustrate our method, table 4 provides an example for a treatment benefit threshold of 5%, that is, if a patient were willing to accept treatment only if it provided a ≥ 5% benefit. In this case, a patient management strategy that bases treatment on the prediction model reduces the number of treatments given by 39% (from 1474 to 889 patients treated) at the cost of a small increase in the number of events (from 472 to 482). Note that the event rate in patients who would not be treated on the basis of prediction is lower than that in the treatment group, suggesting that the prediction model correctly identifies patients at lower than average risk.

**Table 4:** Results for the ACCENT data for a treatment threshold (T) of 0.05.

Strategy	Treat no patients, regardless of model	Treat all patients, regardless of model	Treat according to prediction model		
			Prediction model recommends treatment	Prediction model recommends no treatment	Prediction model total

Number of patients	1397	1474	889	564	1453
Number of events	568 (40.66%)	472 (32.02%)	343 (38.6%)	139 (24.6%)	482 (33.2%)
Decrease in event rate		8.64%			7.49%
Number of treatments		1474 (100%)			889 (61.2%)
Net benefit		8.64% – 100% × 0.05 = 0.0364			7.5% – 61.2% × 0.05 = 0.0443

The results in the first and second columns give the results from the treatment and control groups. The third column shows that there were 889 patients in the treatment group for whom the prediction model estimated a benefit of 5% or more and that 343 of these died. The fourth column shows that there were 564 patients in the control group of the randomized trial with predicted benefit of less than 5%, of whom 139 died. The final column shows the overall results for patients given an individualized prediction.

The size of the benefit is clinically relevant. For example, at a T of 5%, the advantage of prediction compared to treating all patients is 0.008. Now imagine that we had an infallible diagnostic test that could accurately identify 16 out of every 100 patients as entirely cancer-free and not in need of chemotherapy. For a T of 5%, this test would have a net benefit of 16 ÷ 100 × 5% = 0.008. In other words, using the ACCENT model has the same effect as a strategy in which the number of treatments was reduced by 16 per 100 patients and the number of events (recurrences or deaths) was unchanged. We can calculate the reduction in the number of treatments for other levels of T using the formula: decrease in treatment rate = net benefit ÷ T. For T of between 4 and 10%, the use of the prediction model is equivalent to a strategy that reduced the number of treatments given by 12 – 25% without any increase in the number of events.

The use of the prediction model to determine treatment allocation also has the capacity to increase the number of events disproportionately. In table 3, for a small treatment threshold of 2%, the value of -0.007 is the equivalent to a strategy in which an equal number of patients were treated but there was an additional one cancer recurrence or death for every 143 patients. If a patient or physician is willing to accept treatment for a modest benefit, then the risk of not treating, and then having the patient experience an event, is large enough to dictate a strategy of treatment for all patients. In cases where patients would opt for treatment only if the effect size was large, in this case, ≥ 12%, basing individual patient treatment decisions on the prediction model is also on average sub-optimal. Compared to a strategy of treating no patients, prediction-based treatment has results equivalent to a strategy in which 2 – 4% of patients are treated with no reduction in the number of events.

### Moertel trial

The concordance index for the prognostic models in the chemotherapy and no treatment groups were 0.660 and 0.711 respectively, corrected by bootstrapping to 0.626 and 0.687. There was very wide separation in predicted treatment benefit between patients based on the model (from 10% harm to 30% benefit), and this did not appear to result from model miscalibration.

The results of the net benefit calculations are shown in Table 5. The results are broadly similar to the ACCENT results. The model remains of value for higher treatment thresholds compared to ACCENT, presumably because it involves a greater separation of risks. The benefit of prediction is also generally larger than for ACCENT. In this case, estimates of the value of individualized prediction modelling obtained from a single trial were over-optimistic when compared to a later meta-analysis.

**Table 5:** Net benefit for the Moertel trial.

**T**	**Treat all patients**	**Treat by prediction**	**Advantage of prediction**
0.5%	0.133	0.139	0.007
1.0%	0.128	0.132	0.004
2.0%	0.118	0.116	-0.001
3.0%	0.108	0.108	0.000
4.0%	0.098	0.102	0.005
5.0%	0.088	0.091	0.003
7.5%	0.063	0.064	0.001
10.0%	0.038	0.065	0.028
12.5%	0.013	0.035	0.022
15.0%	-0.013	0.029	0.029
20.0%	-0.063	0.021	0.021
25.0%	-0.113	0.011	0.011
30.0%	-0.163	-0.002	-0.002
35.0%	-0.213	0.000	0.000

T is the treatment threshold corresponding to the minimum reduction in absolute risk required to consider opting for treatment. The units are the equivalent of the number of deaths per patient. The "advantage of prediction" column shows the increment in net benefit of prediction compared to the better of "treat all" and "treat none".

### The Dutasteride trials

The concordance index for the model in this dataset was 0.720, corrected by bootstrapping to 0.715. Though the range of risks was extreme – a small number of patients had a predicted benefit less than zero whilst others were predicted to benefit by 45% – over 90% of patients had predicted risk reductions in the range of 0 – 10%.

Net benefit is shown given in Table 6. To interpret this table, we obtained the following estimate of treatment thresholds from a clinician: "80% of patients would want at least a 10% absolute risk reduction; 20% might settle for 5%". There is a small benefit of prediction up to treatment thresholds of 10%. Using the estimates for the distribution of thresholds given above, we estimate that for every 100 patients given an individualized prediction, 10 would elect treatment and one would not experience an event as a result. This is highly cost-effective compared to a strategy of treating all patients, where approximately 30 patients are treated to prevent one event. The benefit of individualized prediction is lost for patients who would require a large treatment effect, presumably because, in this case, the model would need to identify accurately the very small proportion of patients at high risk of an event. Note that, unlike the adjuvant colon cancer case, there is no lower limit to the benefit of prediction. That said, the size of the benefit is very small. For example, for a threshold of 2%, individualized prediction compared to "treat all" is the equivalent of a strategy that reduces the number of treatments by only 1% for an equivalent number of events.

**Table 6:** Net benefit for the Dutasteride data.

**T**	**Treat all patients**	**Treat by prediction**	**Advantage of prediction**
0.5%	0.026	0.027	0.001
1.0%	0.021	0.024	0.002
2.0%	0.011	0.017	0.006
3.0%	0.001	0.011	0.009
4.0%	-0.009	0.012	0.012
5.0%	-0.019	0.009	0.009
7.5%	-0.044	0.002	0.002
10.0%	-0.069	0.002	0.002
12.5%	-0.094	0.000	0.000
15.0%	-0.119	-0.001	-0.001

T is the treatment threshold corresponding to the minimum reduction in absolute risk required to consider opting for treatment. The units are the equivalent of the number of events per patient, where an event is either acute urinary retention or surgical intervention for BPH. The "advantage of prediction" column shows the increment in net benefit of prediction compared to the better of "treat all" and "treat none".

## Discussion

Models to provide individual-level predictions of treatment benefit are becoming widely advocated. We have proposed a method to judge the value of such models, and have applied this method to several real data sets. Our methods appear to be useful for identifying the conditions under which a prediction model is useful. When the group level effect of a treatment is thought to be clinically relevant, prediction modelling can play a role in identifying patients who are unlikely to benefit from treatment. When the effect of treatment is thought to be more limited, prediction modelling may have a role in identifying patients who benefit more than average. However, the use of prediction modelling to guide treatment decisions for a particular patient can be harmful for patients who would accept treatment even if it was of relatively little benefit, as it would recommend no treatment to some patients who would indeed benefit.

In the datasets we have examined, the magnitude of the benefit associated with appropriate clinical implementation of an effective prediction model appears sufficient to warrant development of further models. Models would appear to be particularly appropriate in situations where event rates are low, such as in screening and prevention trials.

Historical trends suggest that prediction models are likely to be of increasing importance. The 1948 streptomycin trial is often considered to be the first randomized trial of the modern era[21]. In this trial there was a very large effect size (an absolute risk reduction of 43%), a high control event rate (92%) and few if any prognostic markers. It is clear that individualized prediction has no role for clinicians using streptomycin to treat tuberculosis. Alternatively, consider a recent randomized trial reporting three-year disease-free survival following fluorouracil plus leucovorin chemotherapy with or without oxaliplatin (which increases toxicity) in patients receiving curative resection for colon cancer[22]. The effect size was small (5% absolute risk reduction in 3-year disease-free survival) and the control event rate was moderate (27%). The trial was very large, with over 2000 patients, which enables the generation of reliable models. This recent trial has many characteristics suggesting that prediction modelling might be of value for the clinician in deciding whether to add oxaliplatin to a patient's treatment regimen.

The exponential increase in research on molecular markers of disease also suggests that prediction modelling will become of increasing importance. One focus of research is the detection of circulating tumor cells as a method of predicting cancer outcome. A recent study demonstrating the value of this approach in breast cancer[23], was accompanied by an editorial[24] in the *New England Journal of Medicine *that emphasized the need for "tools that ... enable us to tailor treatment decisions". Noting that the new assay "permits the prediction of ... survival", the editorial suggests that the findings "may substantially affect ...the standards and practice of decisions about treatment". It has also been suggested that molecular markers could be of benefit in preventive medicine. For example, The American Society of Clinical Oncology has officially recommended that tools be developed to "quantify cancer risk in individuals" with the aim of developing "improved interventions targeting cancer-risk reduction"[25]

We advise appropriate caution in the use of prediction modelling. A statistically significant association between a predictor and a clinical outcome, such as that reported in the study of circulating breast cancer cells, does not demonstrate an advantage to using the prediction tool in clinical management. Similarly, good discrimination and calibration does not establish the clinical value of a prediction tool. Individualized prediction is becoming widely used before there are clear data that it truly improves outcome. With the exception of the ACCENT data analyzed above, most current prediction models, available online [5-7] have yet to be examined to explore their potential impact on eventual patient outcome. Of course a prospective evaluation would be optimal; short of such an effort, we suggest that those developing prediction models use the tools we have developed in this paper. As an immediate first step, we see value in educating users about the possible negative impact of using models to dictate treatment decisions in patients who would opt for treatment even if it was of relatively modest benefit.

## Appendix: A patient's threshold for accepting treatment is equivalent to the ratio between the harm associated with treatment and the harm associated with the event

**Figure 1:**
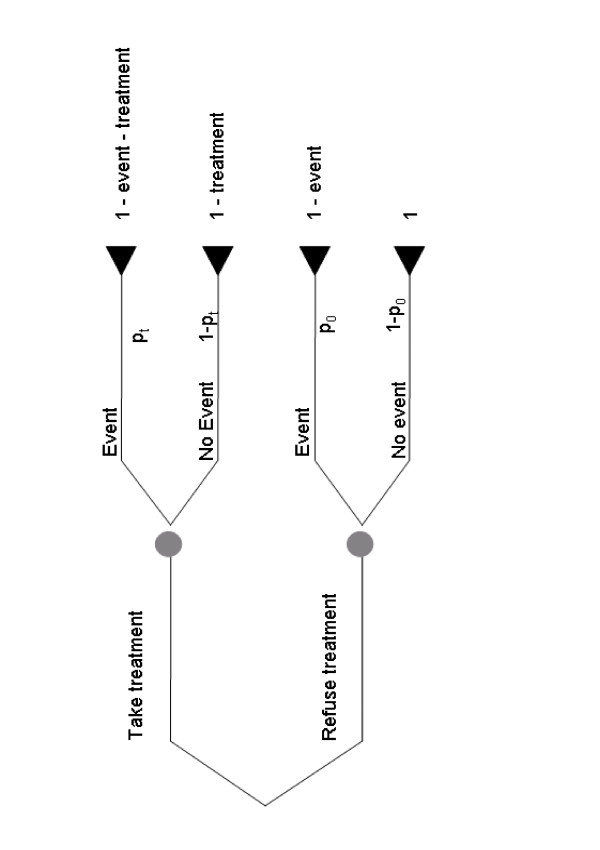
**Decision tree for treatment**.

Figure 1 gives a decision tree for treatment. A patient at risk of event (e.g. recurrence after surgery for breast cancer) can opt for treatment (e.g. chemotherapy) or not, and will experience an event or not. The four possible outcomes – treatment without event; event despite treatment; no event or treatment; event without treatment – are each associated with a certain probability and a certain "value", sometimes assigned by the patient, in terms of, for example, quality adjusted life years, utilities or economic costs. The value of the best possible outcome, avoiding the event without having to go through treatment, is set to one. Experiencing an event and undergoing treatment are each associated with a decrease in the value of a health outcome, the size of which can vary from patient to patient or from clinician to clinician. The probabilities and values for each outcome are multiplied together to determine which decision, treatment or no treatment, leads to the best expected health outcome. In the figure, *p*_*t*_represents the probability of event for patients receiving treatment; *p*_0 _represents the probability of event in untreated patients. "*Event*" is the decrease ("disutility") in how patients value their health if they experience an event; "*treatment*" is the decrease associated with treatment and reflects its inconvenience, side-effects, risks and financial cost.

The model assumes that the disutility of treatment is the same whether or not a patient has an event. This is generally true for the treatments usually incorporated in prediction models, such as chemotherapy, where a treatment causing unpleasant but transient side-effects, is given to try to prevent an event occurring sometime later. The assumption may not hold in certain other situations, such as when treatment causes persistent disutility, and the event is death.

Solving the decision tree, a patient should be uncertain whether to opt for treatment when the expected outcome of treatment and no treatment are equal, that is, when:

*p*_*t *_(1 - *event *- *treatment*) + (1 - *p*_*t*_)(1 - *treatment*) = *p*_0 _(1 - *event*) + 1 - *p*_0_

Expanding and simplifying each side gives:

- *p*_*t*_*event *- *treatment *= - *p*_0_*event*

Rearranging:

p0−pt=treatmentevent
 MathType@MTEF@5@5@+=feaafiart1ev1aaatCvAUfKttLearuWrP9MDH5MBPbIqV92AaeXatLxBI9gBaebbnrfifHhDYfgasaacH8akY=wiFfYdH8Gipec8Eeeu0xXdbba9frFj0=OqFfea0dXdd9vqai=hGuQ8kuc9pgc9s8qqaq=dirpe0xb9q8qiLsFr0=vr0=vr0dc8meaabaqaciaacaGaaeqabaqabeGadaaakeaacqWGWbaCdaWgaaWcbaGaeGimaadabeaakiabgkHiTiabdchaWnaaBaaaleaacqWG0baDaeqaaOGaeyypa0ZaaSaaaeaacqWG0baDcqWGYbGCcqWGLbqzcqWGHbqycqWG0baDcqWGTbqBcqWGLbqzcqWGUbGBcqWG0baDaeaacqWGLbqzcqWG2bGDcqWGLbqzcqWGUbGBcqWG0baDaaaaaa@47B6@

Now *p*_0 _- *p*_*t *_is the absolute risk reduction, in other words, the treatment effect at equipoise, or the threshold we defined in the text as T. *Treatment *is the harm associated with treatment and *event *is the harm associated with the event. Hence T is equivalent to the ratio between harms of treatment and those of an event.

## References

[B1] Gill S, Loprinzi CL, Sargent DJ, Thome SD, Alberts SR, Haller DG, Benedetti J, Francini G, Shepherd LE, Francois SJ, Labianca R, Chen W, Cha SS, Heldebrant MP, Goldberg RM (2004). Pooled analysis of fluorouracil-based adjuvant therapy for stage II and III colon cancer: who benefits and by how much?. J Clin Oncol.

[B2] Loprinzi CL, Thome SD (2001). Understanding the utility of adjuvant systemic therapy for primary breast cancer. J Clin Oncol.

[B3] Kent DM, Hayward RA, Griffith JL, Vijan S, Beshansky JR, Califf RM, Selker HP (2002). An independently derived and validated predictive model for selecting patients with myocardial infarction who are likely to benefit from tissue plasminogen activator compared with streptokinase. Am J Med.

[B4] Rothwell PM, Warlow CP (1999). Prediction of benefit from carotid endarterectomy in individual patients: a risk-modelling study. European Carotid Surgery Trialists' Collaborative Group. Lancet.

[B5] (2006). http://www.mskcc.org/nomograms.

[B6] (2006). http://www.adjuvantonline.com.

[B7] (2006). http://www.mayoclinic.com/calcs/.

[B8] Sonpavde G (2003). Communicating the value of adjuvant chemotherapy. J Clin Oncol.

[B9] Rothwell PM (1995). Can overall results of clinical trials be applied to all patients?. Lancet.

[B10] Glasziou PP, Irwig LM (1995). An evidence based approach to individualising treatment. BMJ.

[B11] Sox H, Blatt M, Marton K (1988). Medical Decision Making..

[B12] Harrell FE, Califf RM, Pryor DB, Lee KL, Rosati RA (1982). Evaluating the yield of medical tests. JAMA.

[B13] Vickers AJ, Elkin EB (2006). Decision curve analysis: a novel method for evaluating prediction models. Medical Decision Making.

[B14] Moertel CG, Fleming TR, Macdonald JS, Haller DG, Laurie JA, Tangen CM, Ungerleider JS, Emerson WA, Tormey DC, Glick JH (1995). Intergroup study of fluorouracil plus levamisole as adjuvant therapy for stage II/Dukes' B2 colon cancer. J Clin Oncol.

[B15] (2006). http://www.mayo.edu/hsr/people/therneau/book/data/colon.html.

[B16] McConnell JD, Bruskewitz R, Walsh P, Andriole G, Lieber M, Holtgrewe HL, Albertsen P, Roehrborn CG, Nickel JC, Wang DZ, Taylor AM, Waldstreicher J (1998). The effect of finasteride on the risk of acute urinary retention and the need for surgical treatment among men with benign prostatic hyperplasia. Finasteride Long-Term Efficacy and Safety Study Group. N Engl J Med.

[B17] Wasson JH (1998). Finasteride to prevent morbidity from benign prostatic hyperplasia. N Engl J Med.

[B18] Roehrborn CG, Boyle P, Nickel JC, Hoefner K, Andriole G (2002). Efficacy and safety of a dual inhibitor of 5-alpha-reductase types 1 and 2 (dutasteride) in men with benign prostatic hyperplasia. Urology.

[B19] Slawin KM, Kattan MW, Roehrborn CG, Wilson T (2006). Development of nomogram to predict acute urinary retention or surgical intervention, with or without dutasteride therapy, in men with benign prostatic hyperplasia. Urology.

[B20] Jansen SJ, Kievit J, Nooij MA, de Haes JC, Overpelt IM, van Slooten H, Maartense E, Stiggelbout AM (2001). Patients' preferences for adjuvant chemotherapy in early-stage breast cancer: is treatment worthwhile?. Br J Cancer.

[B21] MRC Streptomycin in Tuberculosis Trials Committee (1948). Streptomycin treatment of pulmonary tuberculosis. British Medical Journal.

[B22] Andre T, Boni C, Mounedji-Boudiaf L, Navarro M, Tabernero J, Hickish T, Topham C, Zaninelli M, Clingan P, Bridgewater J, Tabah-Fisch I, De Gramont A (2004). Oxaliplatin, fluorouracil, and leucovorin as adjuvant treatment for colon cancer. N Engl J Med.

[B23] Cristofanilli M, Budd GT, Ellis MJ, Stopeck A, Matera J, Miller MC, Reuben JM, Doyle GV, Allard WJ, Terstappen LWMM, Hayes DF (2004). Circulating Tumor Cells, Disease Progression, and Survival in Metastatic Breast Cancer. The New England Journal of Medicine.

[B24] Braun S, Marth C (2004). Circulating Tumor Cells in Metastatic Breast Cancer -- Toward Individualized Treatment?. The New England Journal of Medicine.

[B25] Lippman SM, Levin B, Brenner DE, Gordon GB, Aldige CR, Kramer BS, Garber JE, Hawk E, Ganz PA, Somerfield MR (2004). Cancer Prevention and the American Society of Clinical Oncology. Journal of Clinical Oncology.

